# An Examination of Racial/Ethnic Differences in the Antibiotic Treatment of Community Acquired Pneumonia

**DOI:** 10.1017/ash.2024.127

**Published:** 2024-09-16

**Authors:** Shana Burrowes, Tamar Barlam, Mari-Lynn Drainoni

**Affiliations:** Boston University Chobanian & Avedisian School of Medicine; Boston Medical Center; Boston University School of Medicine

## Abstract

**Background:** Community Acquired Pneumonia (CAP) is the most common reason for antibiotic treatment in hospitalized adults. Some prior studies have found treatment differences by race/ethnicity but research on the topic is limited, results are mixed, and it is unclear if clinical outcomes are affected. We sought to examine whether guideline-concordant CAP care and patient outcomes varied by race/ethnicity. **Methods:** Using the Vizient clinical database, we conducted a cross sectional analysis of all hospitalized patients > = 18 years of age with a primary diagnosis of pneumonia (ICD10 codes: J12-J18) from 2018-2021. Univariate and bivariate analyses examined the distribution of demographic, clinical and hospital characteristics across race/ethnicity. The primary outcome was receipt of therapy concordant with ATS/IDSA Clinical Practice Guideline for CAP. Final models included only patients with bacterial pneumonia and examined the relationship between race/ethnicity and guideline-concordant antibiotic treatment. Secondary analysis examined the interaction between race/ethnicity and concordant antibiotic treatment with length of stay >7 days, 30-day hospital readmission, adverse events or complications in separate models. We used hierarchical multivariable regression models accounting for clustering within patients and among patients hospitalized at the same facility. Due to sample size, significance was assessed with an OR > = 1.2 and p≤ 0.05. All analyses used SAS (v.9.4, SAS Institute Inc. Cary, NC). **Results:** There were 1,277,770 admissions with a primary diagnosis of bacterial CAP. Sixty-nine percent of the sample was White, 18% Black, 8% Hispanic, 2% Asian and 3% identified as other. 56% of the sample received concordant care. In adjusted models Black patients had greater odds of overall concordant care (OR 1.22; p 7 days (OR 0.67 p <.0001), complication or adverse event (OR 0.75 p <.0001), but not readmission within 30 days. **Conclusion:** We observed differences between Black and White patients in the receipt of concordant treatment. Hospital bed size, CMI and region played an important role in both antibiotic treatment decisions and clinical outcomes, indicating that hospital and regional prescribing cultures may play in role in treatment inequities.

